# Climate change, air quality, and respiratory health: a focus on particle deposition in the lungs

**DOI:** 10.1080/07853890.2023.2264881

**Published:** 2023-10-06

**Authors:** Jer-Hwa Chang, Yueh-Lun Lee, Li-Te Chang, Ta-Yuan Chang, Ta-Chih Hsiao, Kian Fan Chung, Kin Fai Ho, Han-Pin Kuo, Kang-Yun Lee, Kai-Jen Chuang, Hsiao-Chi Chuang

**Affiliations:** a School of Respiratory Therapy, College of Medicine, Taipei Medical University, Taipei, Taiwan; b Division of Pulmonary Medicine, Department of Internal Medicine, Wan Fang Hospital, Taipei Medical University, Taipei, Taiwan; c Department of Microbiology and Immunology, School of Medicine, College of Medicine, Taipei Medical University, Taipei, Taiwan; d Department of Environmental Engineering and Science, Feng Chia University, Taichung, Taiwan; e Department of Occupational Safety and Health, College of Public Health, China Medical University, Taichung, Taiwan; f Graduate Institute of Environmental Engineering, National Taiwan University, Taipei, Taiwan; g National Heart and Lung Institute, Imperial College London, London, UK; h JC School of Public Health and Primary Care, The Chinese University of Hong Kong, Hong Kong, China; i Division of Pulmonary Medicine, Department of Internal Medicine, School of Medicine, College of Medicine, Taipei Medical University, Taipei, Taiwan; j Division of Pulmonary Medicine, Department of Internal Medicine, Shuang Ho Hospital, Taipei Medical University, New Taipei City, Taiwan; k School of Public Health, College of Public Health, Taipei Medical University, Taipei, Taiwan; l Department of Public Health, School of Medicine, College of Medicine, Taipei Medical University, Taipei, Taiwan; m Cell Physiology and Molecular Image Research Center, Wan Fang Hospital, Taipei Medical University, Taipei, Taiwan

**Keywords:** Air pollution, asthma, children, COPD, extreme weather, secondary organic aerosol

## Abstract

This review article delves into the multifaceted relationship between climate change, air quality, and respiratory health, placing a special focus on the process of particle deposition in the lungs. We discuss the capability of climate change to intensify air pollution and alter particulate matter physicochemical properties such as size, dispersion, and chemical composition. These alterations play a significant role in influencing the deposition of particles in the lungs, leading to consequential respiratory health effects. The review paper provides a broad exploration of climate change’s direct and indirect role in modifying particulate air pollution features and its interaction with other air pollutants, which may change the ability of particle deposition in the lungs. In conclusion, climate change may play an important role in regulating particle deposition in the lungs by changing physicochemistry of particulate air pollution, therefore, increasing the risk of respiratory disease development.

## Points for Clinical Practice

- Climate change influences particle deposition in the lungs by modifying the physicochemical properties of particulate air pollution, thereby escalating the risk of respiratory disease development.
- It is crucial for healthcare providers to educate patients about the relationship between climate change and respiratory health.
- People with conditions such as asthma, COPD, and allergies must understand how changes in weather, air pollution, and allergens can exacerbate their symptoms.
- Instruction on understanding air quality indices and pollen predictions, along with recommendations on adapting everyday activities and medication regimens in response, is essential.

## 1. Introduction

Climate change is a global challenge that affects various aspects of human health. One of the most significant health impacts of climate change is its effect on air quality and, consequently, respiratory health. Particulate matter (PM) is a key air pollutant associated with respiratory diseases, and particle deposition in the lungs is an essential mechanism through which air pollution affects respiratory health. This review aims to provide a comprehensive understanding of how climate change mediated ambient air quality and respiratory health, especially, focusing on particle deposition in the lungs.

### 1.1. Effects of climate change on urban climate

Climate change influences metropolitan regions in multiple ways, often intensifying existing issues and generating new ones. Urban areas are particularly vulnerable to these consequences due to factors such as dense populations, extensive infrastructure, limited green spaces, and the urban heat island phenomenon. Key impacts of climate change on urban climates include the urban heat island effect, extreme temperature events, air quality, water resources, infrastructure, energy demands, and socio-economic disparities.

The urban heat island effect refers to urban regions experiencing considerably higher temperatures compared to nearby rural areas. This phenomenon results from the prevalence of heat-absorbing surfaces like concrete, asphalt, and buildings. Climate change can amplify the urban heat island effect, resulting in elevated temperatures and heightened health risks for urban inhabitants, including heat stress, heatstroke, and respiratory problems [1]. Moreover, increasing global temperatures lead to more frequent, prolonged, and intense heatwaves. Urban regions are especially susceptible to these events due to the urban heat island effect and the dense concentration of people and infrastructure. Air quality in cities can also be negatively affected by climate change, as it fosters ground-level ozone (O_3_) formation and extends periods of elevated pollution [2]. Rising temperatures and shifting precipitation patterns can also boost the frequency and intensity of wildfires, which emit substantial amounts of particulate matter and other pollutants.

Climate change can induce alterations in precipitation patterns, resulting in more frequent and severe droughts, floods, and storms [3]. These occurrences can place a strain on urban water supplies, infra­structure, and stormwater management systems. Furthermore, heavier rainfall can lead to increased pollutant runoff into water bodies, compromising water quality. Extreme weather events, such as storms, flooding, and heatwaves, can damage urban infrastructure and buildings. Coastal cities also face threats from rising sea levels and coastal flooding. Investing in climate-resilient infrastructure and building designs is crucial for cities to tackle these challenges. Elevated temperatures and more frequent heatwaves can increase energy demand for cooling, burdening urban energy systems and generating more greenhouse gas emissions if the energy is derived from fossil fuels. Lastly, climate change impacts can disproportionately affect low-income and marginalized communities in urban areas. These populations often reside in areas more prone to flooding or extreme heat and have restricted access to resources and infrastructure to help them adapt to and mitigate climate change effects.

### 1.2. Mechanisms for air pollution production by climate change in urban environment

Climate change influences air pollution levels in metropolitan areas through a variety of processes. These processes can modify the generation, dispersion, and elimination of air contaminants, leading to heightened health hazards for city dwellers. Temperature is a primary factor in air pollution production. Elevated temperatures can expedite the formation of secondary air pollutants like ground-level O_3_ [4]. O_3_ is created through a series of chemical reactions between volatile organic compounds (VOCs) and nitrogen oxides (NOx) in sunlight. Increased temperatures can intensify these reactions, resulting in higher O_3_ concentrations in the air. Furthermore, higher temperatures can augment VOC emissions from both natural and human-made sources [5].

Climate change can also modify atmospheric circulation patterns, potentially raising the frequency and duration of stagnant air conditions in urban areas [6]. Stagnation occurs when there is minimal wind, leading to decreased dispersion of air pollutants and causing them to accumulate in localized regions. This can result in elevated concentrations of pollutants like PM and ground-level O_3_. Climate change’s impact on precipitation patterns can lead to alterations in the frequency and intensity of rainfall events [7]. Increased precipitation can help remove air pollutants through wet deposition. Conversely, extended dry periods can result in reduced pollutant removal and increased concentrations of air pollutants in urban regions. Additionally, changes in precipitation can influence the distribution and abundance of biogenic VOC emissions, which can affect air pollution levels.

Climate change can impact vegetation growth and distribution, altering biogenic volatile organic compound (BVOC) emissions, such as isoprene and terpenes [8]. These compounds can react with other pollutants to create secondary pollutants like O_3_ and PM. Warmer temperatures and shifting precipitation patterns can impact BVOC emissions, potentially affecting air pollution levels in urban regions.

The increasing frequency and severity of wildfires due to climate change is a significant air pollution source [9]. Wildfires emit large amounts of PM, along with gases like carbon monoxide (CO) and NOx, which can contribute to O_3_ formation and other secondary pollutants. Human-caused emissions from energy consumption are also linked to climate change, influencing air pollutant production from sources such as power plants, vehicles, and industrial processes [10]. For instance, rising temperatures may lead to a higher demand for air conditioning, resulting in increased emissions from power plants if the energy is derived from fossil fuels.

These processes highlight the intricate relationship between climate change and air pollution production in metropolitan settings. Comprehending and addressing these interactions is essential for devising effective strategies to mitigate air pollution and protect public health in an evolving climate.

### 1.3. Importance of the relationship between climate change, air quality, and respiratory health

Climate change is a pressing worldwide problem with considerable consequences for various aspects of human existence, including health. A major health repercussion of climate change is its influence on air quality and, subsequently, respiratory health [11]. Air contaminants, such as PM, O_3_, and nitrogen dioxide (NO_2_), are linked to heightened morbidity and mortality due to respiratory illnesses [12]. Climate-sensitive results in individuals with pre-existing respiratory conditions contributed to 3,741,705 annual global deaths, with around 74% of these deaths occurring in Asia [13]. According to a previous study, extreme climatic events affected 88% of the global population [14]. In the Asia-Pacific region, 32% of extreme climatic events took place, resulting in 84% of related deaths [14]. The Epidemiology and Impact of COPD (EPIC) Asia population-based survey revealed that chronic obstructive pulmonary disease (COPD) prevalence in Asia-Pacific regions is notably higher than the global average [15]. Furthermore, climate change may pose a greater impact on the health of children, as particle deposition in the airways of an infant or child is higher than that for an adult [16]. Therefore, the impact of climate change and air pollution on human health is a crucial public health concern, particularly in the Asia-Pacific region.

### 1.4. Rationale for focusing on particle deposition in the lungs as a Central mechanism

PM is a primary air contaminant linked to respiratory illnesses, and particle deposition in the lungs plays a vital role in how air pollution impacts respiratory well-being [17]. Comprehending the factors affecting particle deposition, such as particle dimensions, form, and density, as well as lung structure, is crucial in determining the health consequences of air pollution [18]. Moreover, the interplay between climate change and air pollution could result in alterations in particle deposition patterns, potentially aggravating the health effects of air pollution [19]. Therefore, concentrating on particle deposition in the lungs as a key mechanism is essential for understanding the connection between climate change, air quality, and respiratory health.

## 2. Climate change and air quality

### 2.1. Overview of how climate change impacts air quality

Climate change influences air quality *via* several processes, encompassing alterations in temperature, precipitation trends, atmospheric movements, and contaminant emissions [20]. The modification of these elements can contribute to heightened concentrations of critical air pollutants like PM, O_3_, NOx, and VOCs [19]. Additionally, climate change can impact the dispersal and transportation of air pollutants, causing air quality shifts on both local and regional scales [21].

### 2.2. Influence of climate change on PM concentrations, size distribution, and chemistry

Climate change can influence PM concentrations *via* multiple routes, such as more frequent and intense wildfires that generate vast amounts of PM [22]. Moreover, higher temperatures can boost the formation of secondary organic aerosols (SOAs) through the oxidation of VOCs [23]. Alterations in atmospheric movement and precipitation trends due to climate change can also impact the transport, dispersion, and deposition of PM [24]. Climate change can also affect the size distribution and chemistry of PM. For instance, increased temperatures may cause a shift in particle size distribution towards smaller sizes, potentially leading to higher lung deposition. Additionally, climate change could modify the chemical makeup of PM, possibly enhancing its toxicity and health effects [17].

### 2.3. Interaction of climate change with other air pollutants

Climate change can interplay with other air contaminants, such as O_3_, NOx, and VOCs, resulting in intricate effects on air quality. Elevated temperatures can amplify O_3_ formation through intensified photochemical reactions that involve NOx and VOCs [25]. Furthermore, climate change can modify the emissions and atmospheric chemistry of VOCs and NOx, influencing their concentrations and interactions with PM [26]. These interactions between climate change and air pollutants can carry significant consequences for air quality and human well-being. Grasping these intricate relationships is vital for devising efficient approaches to lessen the health impacts of air pollution amid a shifting climate.

## 3. Particle deposition in the lungs

### 3.1. Factors affecting particle deposition

The physical and chemical properties of particles, along with lung structure, impact the process of particle deposition within the lungs. The size of the particles is a significant determinant of where they deposit, with larger particles tending to accumulate in the nasal area, head region, and upper respiratory tract, whereas smaller ones can penetrate deeper into the lungs [27]. Furthermore, the shape of particles can affect deposition, with non-spherical particles showing differing aerodynamic properties compared to spherical ones [28]. The density of particles, which affects how quickly they settle within the respiratory tract, also influences deposition [29]. Additionally, individual variations in factors such as airway dimensions, branching structures, and breathing rates play a role in determining the patterns of particle deposition [30].

### 3.2. Climate change changed ambient particle’s physicochemical properties

Climate change holds substantial sway over alterations in the physical and chemical traits of environmental particles. The ways in which climate change impacts the properties of particulate matter (PM) are multifaceted, shaped by diverse elements such as shifts in temperature, humidity, atmospheric flow, and biological emissions. By adjusting atmospheric processes like nucleation, condensation, and coagulation, climate change can affect the size dispersion of particles. For example, higher temperatures might boost the creation of SOAs *via* the oxidation of VOCs [23]. Newly formed SOAs can contribute to an increase in the number of ultrafine particles, resulting in shifts in the overall size distribution of environmental PM.

Furthermore, climate change can impact the chemical makeup of particles through alterations in emissions, atmospheric chemistry, and deposition processes. Higher temperatures can enhance the release of biogenic VOCs from vegetation, contributing to the formation of SOAs [31]. Also, fluctuations in atmospheric oxidation capacity can cause changes in the chemical makeup of PM, such as the balance between inorganic and organic aerosol components [32].

Climate change can influence the hygroscopic traits of particles. Shifts in temperature and relative humidity can affect the balance of semi-volatile components within particles, leading to changes in particle hygroscopicity [33]. This can then affect the particles’ potential to act as Cloud Condensation Nuclei (CCN), and their overall influence on cloud formation and climate.

With increased temperature, atmospheric ageing processes such as oxidation and heterogeneous reactions on particle surfaces can be accelerated. This can lead to changes in particle composition, morphology, and optical properties, potentially affecting their radiative forcing and health impacts [34]. The mixing state of particles, i.e. the distribution of chemical species within individual particles or particle populations, can be affected by climate change. Shifts in emissions, atmospheric chemistry, and meteorological conditions can lead to fluctuations in the mixing state of particles. These variations can have implications for their radiative properties, CCN activity, and health impacts [35]. In numerous ways, climate change can alter the physical and chemical properties of environmental particles, including their size distribution, composition, hygroscopicity, and mixing state. These changes can have implications for air quality, climate, and human health.

### 3.3. Mechanisms of particle deposition in the lung

Three main processes govern the deposition of particles in the lungs: impaction, sedimentation, and diffusion [36,37]. Impaction is a process whereby the inertia of a particle makes it proceed straight ahead rather than following the airflow as it bends around an airway’s curve. This phenomenon mainly impacts larger particles, typically over 5 µm in diameter. These particles then strike the wall of the airway and accumulate in the upper respiratory tract, encompassing the trachea, bronchi, and bronchioles. Impaction is more probable during periods of high airflow rates, such as during fast or deep inhalation.

Sedimentation is the process by which particles settle due to gravity and is more efficient for particles with diameters between 1 and 5 µm. Sedimentation becomes more prominent during slow and shallow breathing, as particles have additional time to settle in the airways. It primarily affects the deposition of particles in the lower respiratory tract, which includes the bronchioles and alveoli.

Diffusion is the leading mechanism for the deposition of ultrafine particles, typically less than 0.5 µm in diameter. These particles undergo Brownian motion, which results in random movements due to collisions with air molecules. Consequently, ultrafine particles can diffuse to the walls of the airways and deposit in the alveolar region of the lungs. Diffusion is more efficient at low airflow rates and decreases in significance as particle size grows.

In conclusion, the forces that dictate particle deposition in the lungs depend on the particle’s size, with impaction being the primary mechanism for larger particles, sedimentation for medium-sized particles, and diffusion for ultrafine particles. These mechanisms are subject to influence by various factors, such as the morphology of the lung, breathing patterns, and the geometry of the airway.

### 3.4. Effects of climate change on particle deposition in the lungs

Climate change could have a substantial indirect impact on the deposition of particles in the lungs. While the precise effects of climate change on particle deposition in the lungs remain to be fully elucidated, it’s indisputable that climate change influences air quality, and consequently, respiratory health. Here are a few potential ways in which climate change might impact particle deposition in the lungs.

Firstly, climate change can result in heightened concentrations of PM in the atmosphere due to more frequent and intense wildfires, drier conditions fostering dust storms, and altered patterns of atmospheric circulation [38]. Elevated PM concentrations could lead to increased particle deposition in the lungs. In addition, PM size distribution, which is modified by climate change, affects the deposition patterns of particles in the respiratory system. For instance, a higher occurrence of wildfires due to climate change can elevate the proportion of ultrafine particles in the atmosphere, thereby increasing the amount of these particles that can deeply penetrate the lungs and deposit in the alveolar region.

Thirdly, climate change can impact the chemical composition of air pollutants. Variations in temperature, humidity, and precipitation can influence the formation and transformation of secondary pollutants, such as sulfate and nitrate particles [38]. These shifts in pollutant chemistry could alter the patterns of particle deposition in the lungs and affect respiratory health.

Climate change can also lead to increased pollen production, thanks to longer growing seasons, earlier flowering times, and higher concentrations of CO_2_ [39]. Pollen grains and their submicron components can be inhaled into the bronchi and tracheal regions of the lung. Climate change can also influence the interaction between PM and other air pollutants, such as O_3_ and VOCs. These interactions may modify the properties of PM, thereby affecting its deposition in the lungs.

In conclusion, climate change can affect particle deposition in the lungs by influencing air quality, particularly in terms of particulate matter concentrations, size distribution, and chemical composition. These changes could potentially lead to adverse health effects, especially for vulnerable groups like individuals with pre-existing respiratory conditions, the elderly, and children.

### 3.5. Removal mechanisms for inhaled particle in the lungs

The elimination processes for particles inhaled into the lungs largely depend on factors such as the particle’s size, shape, and chemical characteristics, as well as the individual’s lung structure and respiratory function. The predominant methods for removing inhaled particles from the lungs include mucociliary clearance, alveolar macrophage phagocytosis, and lymphatic system clearance.

Mucociliary clearance is the primary process that eliminates inhaled particles from the upper respiratory tract, encompassing the trachea, bronchi, and bronchioles [40]. The respiratory epithelium is covered by cilia and blanketed by a mucus layer. The coordinated movement of the cilia propels the mucus layer (which traps particles) toward the throat, where it is either swallowed or coughed up. This method is particularly efficient at removing larger particles, though it is less effective with smaller particles [41].

In the lower respiratory tract (namely the alveoli), alveolar macrophages are instrumental in particle removal. These immune cells identify and engulf particles through a process known as phagocytosis. Once the particles are inside the macrophage, they are typically disassembled by enzymes or confined in compartments referred to as phagolysosomes. These macrophages, carrying the engulfed particles, can then migrate toward the mucociliary escalator or the lymphatic system for expulsion [42].

Furthermore, the lymphatic system plays a significant role in eliminating inhaled particles, especially those small enough to penetrate deeply into the lung tissue. These particles can traverse through the interstitial spaces and be absorbed by the lymphatic vessels, which ultimately drain into the bloodstream. Once in the bloodstream, the particles can be expelled from the body through the liver, kidneys, or gastrointestinal tract [43].

In summary, the processes that remove inhaled particles from the lungs are intricate and multifaceted, with the effectiveness of each mechanism being contingent on the properties of the particles and the individual’s respiratory functionality.

### 3.6. Health implications of particle deposition in the lungs

Inhalation and subsequent deposition of particles in the lungs can result in a range of health outcomes, including inflammation, oxidative stress, and particle migration into the circulatory system. Once inhaled, these particles can stimulate local inflammation by triggering pulmonary immune cells like macrophages and neutrophils, resulting in the release of pro-inflammatory cytokines and chemokines [44]. Another significant way in which particles can affect lung health is through oxidative stress. Particles can stimulate the creation of reactive oxygen species (ROS), which can inflict damage on cellular components like lipids, proteins, and DNA, thus playing a role in the onset of respiratory diseases [45]. In addition to these effects, ultrafine particles are small enough to pass from the lungs into the circulatory system, potentially inciting systemic inflammation and contributing to the development of cardiovascular diseases [46].

## 4. Actions and preparation for climate change

It’s essential for medical professionals to inform patients of the links between climate change and respiratory health. Individuals with conditions like asthma, COPD, and allergies need to be aware of how meteorological shifts, air pollution, and allergens can aggravate their symptoms. This instruction might include guidance on understanding air quality indices, pollen projections, and how to modify their day-to-day activities or medication schedules in response.

Practitioners ought to have strategies ready to deal with possible surges in patient numbers during periods of bad air quality or heatwaves. This might entail ensuring an adequate supply of necessary medicines, arranging for additional staff, and implementing telemedicine facilities to offer remote care when needed. Moreover, it’s crucial to counsel patients on protective measures during times of adverse air quality or excessive heat. Suggestions might include staying indoors with windows shut, using air conditioning or air purifiers, wearing protective eyewear to shield against heightened allergens, and maintaining hydration.

Medical practitioners can have a significant influence in policy-making. Advocacy for policies that alleviate climate change, cut air pollution, and invest in cleaner, renewable energy sources can have a key role in safeguarding respiratory health amid climate change. Keeping updated with the most recent research on the impacts of climate change on respiratory health is vital. Forming collaborations with scientists, public health professionals, and other stakeholders to drive further research in this domain is a beneficial approach.

The potential psychological and societal impacts of climate change on patients should also be considered. Certain individuals may experience stress or anxiety due to climate change, which could potentially intensify respiratory symptoms. In some situations, referrals to mental health specialists may be appropriate. Patients should be encouraged to help mitigate the effects of climate change through lifestyle modifications, such as lowering energy use, recycling, and utilizing active or public transport. Not only can these actions diminish an individual’s carbon footprint, but they can also confer additional health advantages.

Embedding these strategies into respiratory clinical practice can lead to a more preemptive and holistic approach to patient care in the climate change era.

## 5. Conclusion

**Figure 1.**
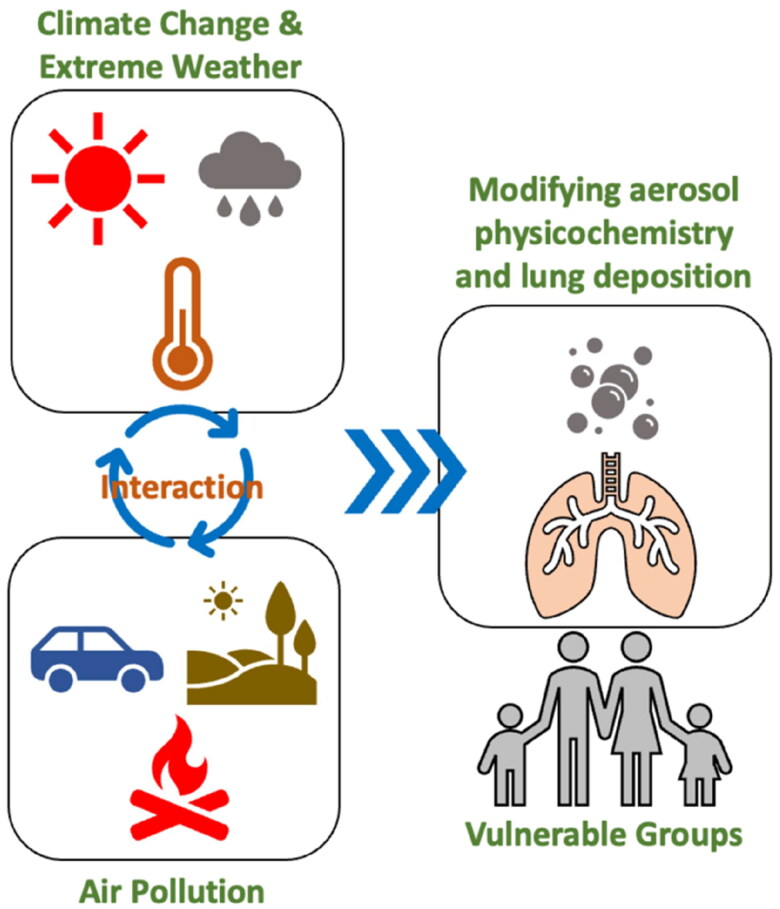
Interplay between climate change, air quality, and respiratory health, with specific emphasis on the deposition of particulates in the lungs. Climate change has the potential to heighten the severity of air pollution and alter the size, spread, and chemical composition of particulates. These changes can in turn impact their deposition in the lungs and corresponding health effects. Respiratory health conditions such as asthma, COPD, infectious disease and lung cancer in adults and children, can be especially influenced by these alterations in air quality under climate change.

This review has underscored the intricate interplay between climate change, air quality, and respiratory health, with specific emphasis on the deposition of particulates in the lungs as shown in Figure 1. Climate change has the potential to heighten the severity of air pollution and alter the size, spread, and chemical composition of particulates. These changes can in turn impact their deposition in the lungs and corresponding health effects. Respiratory health conditions such as asthma, COPD, infectious disease and lung cancer in adults and children, can be especially influenced by these alterations in air quality under climate change. Future research should aim at bridging current knowledge gaps, investigating emerging fields of research, and crafting innovative methods for studying particle deposition and respiratory health by climate change.

## Competing interests

The authors declare that they have no conflicts of interest.
